# Comprehensive review of surgeries for obstructive sleep apnea syndrome

**DOI:** 10.5935/1808-8694.20130139

**Published:** 2015-10-08

**Authors:** Macario Camacho, Victor Certal, Robson Capasso

**Affiliations:** a MD (Consulting Assistant Professor Department of Otolaryngology - Head and Neck Surgery Sleep Surgery); b MD (Department of Otorhinolaryngology, Hospital São Sebastiao, Sta Maria da Feira, Portugal CINTESIS - Center for Research in Health Technologies and Information Systems, University of Porto, Portugal); c MD (Assistant Professor Department of Otolaryngology - Head and Neck Surgery Sleep Surgery Division Stanford Hospital and Clinics, Stanford, CA, USA); d Stanford Hospitals and Clinics, Stanford, CA

**Keywords:** sleep apnea, obstructive, sleep apnea, obstructive/surgery, snoring, therapeutics

## Abstract

There are several surgical treatment modalities utilized for obstructive sleep apnea syndrome (OSAS). OSAS can cause excessive daytime sleepiness as well as cardiovascular morbidity and mortality. Patients who fail medical management often seek surgical treatment.

**Objective:**

This paper reviews surgical treatment options for obstructive sleep apnea syndrome to include original descriptions as well as outcomes for snoring, apnea-hypopnea indices, and mortality benefits.

**Method:**

A literature review was performed for OSAS surgical treatment options for soft tissue and skeletal surgeries. Articles with the original descriptions and surgical reviews are included for each procedure.

**Results:**

A total of twenty-eight surgical treatment modalities for OSAS were identified. Original article authors and year of description were obtained and presented. Polysomnographic data for apnea indices, apnea-hypopnea indices and mortality are presented.

**Conclusion:**

There is a large amount of variability in outcomes for sleep surgeries, however, in order to maximize success and cure rates, multiple procedures are most often necessary. Sleep surgeons must get familiar with modern surgical concepts and techniques, and participate in multi-disciplinary care in order to maximize treatment outcomes.

## INTRODUCTION

Obstructive sleep apnea syndrome (OSAS) manifests with excessive daytime sleepiness and affected patients can be treated either medically or surgically^1^. Continuous positive airway pressure (CPAP) was first described by Sullivan et al. in 1981 as highly efficacious treatment for OSAS^2^. Although CPAP has high efficacy, the effectiveness of the therapy is limited, as demonstrated by 46%-83% of patients being non-adherent to therapy (defined as > 4 hours of use per night)^3^. Patients who fail medical management often seek surgical treatment options. There is debate as to the effectiveness of surgical treatment for OSAS. Surgical treatment, guided by proper patient selection, counseling and realistic expectations increases the likelihood of success and patient satisfaction, respectively. The goal of this paper is to review surgical approaches for OSAS treatment.

## METHOD

A literature review was performed for adult obstructive sleep apnea syndrome surgical treatment. Titles and abstracts were reviewed to identify surgical treatment modalities. The original study describing each procedure, the studies that contributed significantly and systematic reviews are included in this article. Surgical techniques are described based on sub-site and in the order that they first were described in the literature as treatment for obstructive sleep apnea. The search was performed on MedLine from inception to June 2013. The original articles providing the description of each procedure are presented from the earliest being uvulopalatopharyngoplasty (UPPP) in 1964, to the most recent, which is transoral robotic surgery (TORS) for tongue base reduction in 2010.

Subsequently, the articles describing outcomes for each of the procedures were also reviewed. Articles that contributed substantially were reviewed and were included. As a literature review, this study is exempt from the Stanford IRB protocol review.

## RESULTS

The results of the MedLine search yielded the following surgical modalities: uvulopalatopharyngoplasty, tracheostomy, tonsillectomy, mandibular advancement, genioglossus advancement, hyoid suspension, cautery assisted uvuloplasty, maxillomandibular advancement (MMA), laser assisted uvuloplasty (LAUP), midline glossectomy, arytenoid reduction, epiglottectomy, epiglottopexy, epiglottoplasty, transpalatal advancement pharyngoplasty (TAP), cautery-assisted uvulopalatoplasty, cautery-assisted palatal stiffening operation (CAPSO), mandibular distraction, radiofrequency ablation of the soft palate, rapid maxillary expansion (RME)/surgically assisted rapid palatal expansion (SARPE), radiofrequency ablation of the tongue, tongue stabilization/tongue suspension, hypoglossal nerve stimulator/implant (HGNS), nasal surgery, lateral pharyngoplasty, palatal implants, Z-palatoplasty (ZPP), tongue base coblation, submucosal minimally invasive lingual excision (SMILE), expansion sphincter pharyngoplasty and transoral robotic surgery (TORS).

Table 1 chronologically presents surgeries as they appeared on MedLine or the searched articles' references for the original procedural description. In addition, a total of 11 other articles which are systematic reviews, meta-analyses or comprehensive overviews are also included in this review1., 4., 5., 6., 7., 8., 9., 10., 11., 12., 13., 14..

**Table 1:** The evolution of sleep apnea surgeries. Listed chronologically based on a MedLine search and references from those searches.

Surgical Modality as Treatment for OSAS in Adult Patients	Year described in MedLine (or references)	Authors
	1964 (Snoring)	Ikematsu^15^
Uvulopalatopharyngoplasty	1960 (OSAS-abstract)	Conway, Fujita, Zorick^16^
	1981 (OSAS-article)	Fujita, Conway, Zorick, Roth^17^
Tracheostomy/Tracheotomy	1960	Valero & Alroy^16^
Tonsillectomy	1975	Sussman, Podoshin, Alroy^19^
Mandibular advancement	1979	Kuo, West, Bloomquist^20^
Genioglossus advancement	1984	Riley, Guilleminault, Powell^21^
Hyoid suspension	1984	Riley, Guilleminault, Powell^21^
Maxillomandibular advancement (MMA)	1986	Riley, Powell, Guilleminault, Nino-Murcia^22^
		Snoring: 2 groups: 1) Kamami^23^, and 2)
	1990 (Snoring)	Haraldsson and Carenfelt^24^
Laser assisted uvuloplasty (LAUP)	1994 (OSAS)	OSAS: Kamami^25^
Midline glossectomy	1991	Fujita, Woodson, Clark, Wittig^26^
Aryepiglottic fold reduction	1991	Fujita, Woodson, Clark, Wittig^26^
Epiglotteetomy	1991	Fujita, Woodson, Clark, Wittig^26^
Nasal surgery	1992	Series, St. Pierre, Carrier^27^
Transpalatal Advancement Pharyngoplasty (TAP)	1993	Woodson, Toohill^28^
		Snoring: Blythe, Henrich, Pillsbury^29^
	1995 (Snoring)	
Cautery-assisted uvulopalatoplasty/Cautery-assisted palatal stiffening operation (CAPSO)	2000 (OSAS)	OSAS: 2 groups: 1) Wassmuth, Mair, Loube, Leonard^30^ and 2) Mair & Day^31^
Epiglottopexy/Epiglottoplasty	1998	Chabolle, Wagner, Sequert, Lachiver, Coquille, Fleury, Blumen^32^
Radiofrequency ablation of the soft palate	1998	Powell, Riley, Troell, Li, Blumen, Guilleminault^33^
Rapid maxillary expansion (RME)/Surgically-assisted rapid palatal expansion (SARPE)	1996 (Case report)	Case report: Palmisano, Wileox, Sullivan, Cistulli^34^
	1998 (Case series)	Gistulli^34^Case series: Cistulli & Palmisano^35^
Radiofrequency ablation of the tongue	1999	Powell, Riley, Guilleminault^36^
Tongue stabilization/Tongue Suspension	2000	DeRowe, Gunther, Fibbi, Lehtimaki, Vahatalo, Maurer, Ophir^37^
Hypoglossal nerve stimulator/implant (HGNS)	2001	Sehwartz AR, Bennett ML, Smith PL, De Backer W, Hedner J, Boudewyns A, Van de Heyning P, Ejnell H, Hochban W, Knaack L, Podszus T, Penzel T, Peter JH, Goding GS, Erickson DJ, Testerman R, Ottenhoff F, EiseleDW^38^
Mandibular distraction	2002	Li, Powell, Riley, Guilleminault^39^
Lateral pharyngoplasty	2003	Cahali^40^
Palatal implant	2004	Ho, Wei, Chung^41^
Z-palatoplasty (ZPP)	2004	Friedman, Ibrahim, Vidasagar, Pomeranz, Joseph^42^
	2005	He, Chen, Jin, He, Su, Fan, Yu^43^
Tongue base coblation	2006 (With mierolaryngoseopy)	Robinson, Ettema, Brusky, Woodson^44^
	2006 (With endoscopes)	Maturo & Mair^45^
Submucosal minimally invasive lingual excision (SMILE)	2006 (Pediatrics)	Maturo & Mair^46^
	2008 (Adults)	Friedman, Soans, Gurpinar, Lin, Joseph^47^
Expansion sphincter pharyngoplasty	2007	Pang, Woodson^48^
Transoral Robotic Surgery (TORS)	2010	Vicini, Dallan, Canzi, Frassineti, La Pietra, Montevecchi^49^

## Nasal surgeries

Series et al.^27^ described 20 adults in 1992 who had undergone nasal surgeries, with significant improvement in nasal resistance, but minimal improvement in the respiratory disturbance index (RDI) from 39.8 ± 6.1/h to 36.8 ± 5.9/h. A recent meta-analysis by Li et al.^10^, analyzing nine articles with pooled AHI results demonstrating a decrease from 35.2 ± 22.6/h to 33.5 ± 23.8/h, with a total success rate of 16.7%. Although nasal procedures do not have a statistically significant improvement in isolated treatment for OSAS, they do improve nasal breathing and can help improve CPAP compliance and reduce CPAP pressures from a mean of 11.9 down to 9.2 centimeters of water pressure^50^.

## Palatal surgeries

### Uvulopalatopharyngoplasty (UPPP)

Ikematsu et al. first began performing uvulopalatopharyngoplasties in 1952, and subsequently published his results of the procedure as treatment of snoring in 196415., 51.. Over the years, modifications have been described40., 42., 52.. Initial techniques described removal of larger amounts of palatal tissue and subsequently had increased risks for foreign body sensation, dry throat, globus sensation, lower fundamental frequency of speech, problems with phlegm, velopharyngeal insufficiency and nasopharyngeal stenosis53., 54., 55., 56.. However, newer descriptions have been modified in a manner that re-organizes and preserves tissue^52^. Systematic reviews of UPPP surgeries have demonstrated wide variability among polysomnography results. Caples et al.^4^ pooled data from 15 studies demonstrating a pre-operative AHI of 40.3/h, and post-operative AHI of 29.8/h for a 33% reduction. Elshaug et al.^6^ pooled seven studies and demonstrated a 51.5% success rate in providing a 50% reduction in AHI and/or ≤ 20/h^6^. Franklin et al.^7^ assessed complications and found 30 deaths in six studies between 1989 and 2004.

### Laser assisted uvuloplasty (LAUP)

Stiffening of the palate was initially described as treatment for snoring, and the procedure was subsequently applied as treatment for sleep apnea23., 25.. Kamami^23^, then Haraldsson & Carenfelt^24^ reported their initial experiences with LAUP in 1990 as a treatment modality that is more easily performed under local anesthetic.^,^ Caples et al.^4^ assessed two randomized controlled trials and six observational studies, with a pooled decrease of 32% in the AHI. Elshaug et al.^6^ demonstrated a pooled success rate of 48.8% (confidence interval (CI) of 37.6–60). One issue with the LAUP is that patients often require several treatments, Kamami23., 25. described that on average patients were treated with 4 individual sessions.

### Transpalatal advancement pharyngoplasty (TAP)

Because the results of UPPP procedures were inconsistent, Woodson & Toohill^28^ developed the TAP as a way to advance the palate anteriorly more consistently. The technique involves a gothic arch incision from posterior to the alveolus with a continuation forward in the palatoglossal fold, the hamulus is exposed and fractured, the posterior hard palate is removed, redundant mucosa is excised and lateral flaps are advanced and sutured to the alveolar mucoperiosteum. The response from the initial study in 6 patients demonstrated a mean decrease from 69.3 ± 32.1/h to 26.6 ± 25.3/h^28^. Shine & Lewis^53^ described a modification of the TAP, which utilizes a “propeller incision” which has a lower incidence of oronasal fistula. Shine & Lewis^54^ compared patients who underwent the traditional gothic arch incision compared to the newer propeller incision and found that there was a 31% improvement in success rate with the propeller incision.

### Cautery-assisted uvulopalatoplasty/Cauteryassisted palatal stiffening operation (CAPSO)

Initially described as a simple, inexpensive single stage clinic procedure for snoring, the procedure was subsequently described as treatment for OSAS29., 30., 31., 55.. When performed for snoring treatment, Blythe et al.^29^ describe that 83% of spouses reported either resolution or significant improvement in the patients' snoring. Mair & Day^31^ reported treating 200 consecutive patients over an 18-month period with success rates of 92% initially, and a slight decrease in success down to 77% after 1 year. Wassmuth et al.^30^ demonstrated a decrease in AHI from 25 ± 12.9/h to 16.6 ± 15.0/h (*p* = 0.01). Pang and Terris'^55^ modification involves performing an uvulectomy, removing a horizontal strip of mucosa from the anterior aspect of the soft palate and the creation of vertical trenches in the soft palate bilaterally. The modified CAPSO reports an improvement in their patients' AHI from 12.3/h to 5.2/h (*p* < 0.05).

### Radiofrequency ablation (RFA) of the soft palate

RFA of the soft palate was described by Powell et al.^33^ in a group of 22 healthy men in 1998. This initial study demonstrated that the procedure was effective for the treatment of snoring (snoring scores decreased by 77%), an improvement in AHI and only mild patient discomfort. Caples et al.^4^ reviewed seven observational studies and showed a mean change in AHI from 23.4/h to 14.2/h. Elshaug et al.^6^ demonstrated a 60% success rate in the two studies evaluated (95% CI of 37.6–60).

### Lateral pharyngoplasty

Cahali^40^ described treatment of OSAS by performing a bilateral tonsillectomy, with elevation of the superior pharyngeal constrictor muscle within the tonsillar fossa, superior traction is placed on the upper palatopharyngeus muscle, and a palatine flap is created laterally, a subtotal resection of the palatopharyngeus muscle is made and the flaps are closed in a z-plasty fashion. Results demonstrated an improvement in AHI from 41.2/h to 9.5/h (*p* = 0.009).

### Palatal implants

There was one double-blinded, randomized, placebo-controlled trial which demonstrated a modest reduction in AHI, from 23.8/h to 15.9/h for the palatal implant arm *vs*. 20.1/h to 21.0/h for the placebo group, demonstrating a statistically significant difference in the two groups, *p* < 0.001^56^. Choi et al.^57^ pooled data from seven studies and demonstrated a statistically significant reduction in AHI with and a standard mean difference of -0.378; 95% CI of 0.619 to -0.138, *p* = 0.02. The meta-analysis data also demonstrated a 9.3% extrusion rate.

### Z-palatoplasty (ZPP)

The ZPP was described by Friedman et al.^42^ for treatment of patients with OSAS who have previously undergone a tonsillectomy. In this technique, the mucosa over the anterior soft palate and uvula are removed, exposing the underlying muscle, followed by bisection of the uvula and inferior soft palate with a cold knife, the flaps are then rotated laterally over the soft palate and are sutured into position. This study demonstrated improved results compared to standard UPPP (25 patients in each arm), with ZPP decreasing AHI from 41.8 ± 26.4/h down to 20.9 ± 19.3/h.

### Expansion sphincter pharyngoplasty

Pang & Woodson^48^ describe a technique in which a horizontal incision is made in the palatopharyngeus muscle (after tonsillectomy), superolateral incisions are made on the soft palate, in the inferior aspect of the palatopharyngeus muscle is then suspended superolaterally. The 45 patients had a BMI < 30 kg/m^2^, were Friedman stage 2 or 3, Type 1 Fujita and had lateral pharyngeal wall collapse, with an AHI improvement from 44.2 ± 10.2/h to 12.0 ± 6.6/h (*p* < 0.005).

## Tonsillectomy

Whereas, in children there is a significant amount of data; in adults, tonsillectomy studies as treatment for OSAS is limited. Sussman et al.^19^ described performing a tonsillectomy in two obese patients who also had significant tonsillar hypertrophy, and had resolution of their sleepiness after the tonsillectomy. One study by Stow et al.^58^ of 13 patients who underwent tonsillectomy (11 with additional nasal surgeries) as treatment for OSAS and found a significant reduction in the AHI from 31.7/h to 5.5/h (*p* = 0.0002). The effectiveness of this procedure is dependent on several factors to include: the anatomy of the patients, the BMI, neck circumference, tongue size, and the anatomical location of the apneic obstruction during sleep.

## Tongue

### Radiofrequency ablation (RFA) of the tongue

After radiofrequency ablation of the palate had successful outcomes, Powell et al.^36^ piloted a study on 18 patients with sleep disordered breathing to assess the effectiveness for the RFA to the tongue in 1999. This study found a mean reduction in AHI from 39.6/h to 17.8/h, with no change in speech or swallowing, and only one infection. Kezirian & Goldberg^9^ reviewed 11 studies on results of RFA of the tongue and demonstrated a wide range of AHI outcomes, ranging from 20% to 83% successful. Most studies demonstrate decreased daytime hypersomnolence, improved quality of life and improvement in the lowest oxygen saturation.

### Tongue stabilization/Tongue suspension

DeRowe et al.^37^ described the Repose system in 2000 as a new, minimally invasive procedure in which there is suspension of the base of tongue to reduce tongue-base collapse. This initial study on 16 patients demonstrated a mean RDI improvement from 35/h to 17/h (*p* = 0.001) and 14 out of 16 bed-partners reported improvement in snoring. Kezirian & Goldberg^9^ reviewed six studies and found 20% to 57% success rates.

### Hypoglossal nerve stimulation

Investigations regarding the physiology of the genioglossus muscle and electrophysiological stimulation began with Guilleminault et al.^59^ in 1978. Hypoglossal nerve stimulation studies were later continued by a few groups to include: 1) Fairbanks & Fairbanks^60^ in 1993, and 2) Eisele et al.^61^ in 1997. In 2001, Schwartz et al.^38^ described the pilot study for placement of an implantable device which provides electrical stimulation of the hypoglossal nerve as treatment for OSAS. In this study, a total of eight patients with a mean AHI of 52 ± 20.4/h in NREM and 48.2 ± 0.5/h in REM, demonstrated a decrease down to 22.6 ± 12.1/h in NREM and 16.6 ± 17.1/h in REM (*p* < 0.001).^38^ The current procedure involves placement of an implantable pulse generator, with a respiratory pressure sensor and a stimulation lead, which stimulates the hypoglossal nerve during sleep^62^.

### Tongue base coblation

He et al.^43^ studied UPPP with tongue base coblation in 112 patient with OSAS and at 12 months post-operatively and demonstrated 24 patients being cured (21.4%), 52 patients had notable improvement (46.4%), 16 had some improvement (14.2%), and 20 patients had no effect. Robinson et al.^44^ described lingual tonsillectomies in 18 patients with massive (N = 10) and modest (N = 8) lingual tonsillar hypertrophy, with moderate pain (7–10 days), a mean of 20 mL of blood loss during the procedure and only two patients requiring revision. Robinson et al.^44^ described using microlaryngoscopy with an operating microscope during the procedure, while Maturo & Mair^45^ in a letter to the editor described coblation lingual tonsillectomy utilizing a rubber bite block and a 70 degree sinus endoscope placed trans-orally, which is the current technique used in many institutions.

### Submucosal minimally invasive lingual excision (SMILE)

Maturo & Mair^46^ described this technique for use in children with good results. The first article to evaluate the effectiveness in adults was Friedman et al.^47^, in 2008, in which they demonstrated a 64.6% success rate *(p* = 0.024), however, there was a higher necessity for narcotic pain control (3.8 ± 3.8 days *vs*. 2.4 ± 3.5 days) and a longer time to return to a normal diet (4.9 ± 4.5 days *vs*. 2.9 ± 4.1 days) when compared to patients who underwent radiofrequency ablation of the tongue, respectively.

### Transoral robotic surgery (TORS)

In 2010, Vicini et al.^48^ described their outcomes utilizing TORS for OSAS. The study reported on 10 patients with a mean preoperative AHI of 38.3/h which decreased to 20.6/h with surgery of the tongue base and epiglottis using the transoral robotic surgery^63^. Additional studies have demonstrated the effectiveness of TORS for OSA, such as the Friedman et al.^64^ 2012 publication, which demonstrated a reduction in AHI from 54.6/h to 18.6/h (*p* < 0.001) and a success rate of 66.7% and cure rate of 18%.

## Hyoid suspension

Resistance of airflow is directly proportional to the length of the airway, therefore, a decrease in airway length will provide a decrease in resistance. The decrease in airway length can be accomplished by performing a hyoid suspension, which was described in 1984 by Riley et al.^21^. Kezirian & Goldberg^9^ evaluated 4 studies' data and found that when the hyoid suspension is performed at the same time as another surgery, then there are better outcomes with a 71% successful rate, however, when performed in isolation after previous unsuccessful surgery, then there is a 35% success rate.

## Multiple procedures

Because obstructive apneic events during sleep occur in multiple locations in the airway, the treatment options are therefore more successful if those multiple areas of obstruction are addressed, rather than performing single site surgery. A common anatomical site of obstruction is the hypopharyngeal and supraglottic region, therefore, removal or reduction of tissue in this area can help reduce obstructive events. Fujita et al. described the midline glossectomy in 12 patients with simultaneously performed laser lingual tonsillectomy (N = 7), reduction in aryepiglottic folds (N = 10), partial epiglottectomy (N = 5) and all received a temporary tracheostomy.^26^ Fujita et al.^26^ results demonstrated an RDI decrease from 60.6/h to 14.5/h in responders (42%) and a decrease from 62.6/h to 48.4/h in non-responders (N = 7). As part of this comprehensive review, no studies were identified for isolated aryepiglottic fold reduction or isolated epiglottectomy as treatment for OSAS. Kezirian & Goldberg^9^ reviewed five studies in which multiple procedures were performed and demonstrated a range of success (25%-83%).

Lin et al.^11^ pooled 58 studies in which multiple procedures were performed, with 1,978 patients, mean age of 46.2 years, with a pre-operative AHI of 48/h and found a 60.3% reduction in the AHI, which corresponds to a 66.4% success rate.

## Skeletal surgeries

### Mandibular advancement

Kuo et al.^20^ first described the mandibular advancement surgery in 1979 as treatment for hypersomnia sleep apnea (HSA), which is now called obstructive sleep apnea syndrome (OSAS). They described three cases of traumatically induced mandibular retrognathism which caused sleep apnea with daytime hypersomnia, and each had resolution after the mandibular advancement^65^. It is the mandibular advancement procedure which was the groundwork for future advancement of the maxillomandibular complex in patients who did not have retrognathia, as treatment for OSAS.

### Genioglossus Advancement/Genial Tubercle Advancement (GTA)

This procedure is often combined with other procedures such as a hyoid suspension, or uvulopalatopharyngoplasty. Initially described by Riley et al.^21^, in combination with the hyoid suspension as a new treatment for OSAS in a 24 year old male with a BMI of 36.1 kg/m^2^ who had failed previous tonsillectomy and septoplasty. Kezirian & Goldberg^9^ demonstrated a pooled GTA success rate of > 60%, with the AHI decreasing from 60/h to 29/h. Two key factors influencing the outcomes were preoperative BMI and AHI; demonstrating that those with a BMI < 30 kg/m^2^ having a 64% success rate compared with BMI > 30 kg/m^2^ having a 41% success rate. A preoperative AHI < 50/h was successful in 71% of patients, while those with an AHI > 50/h was successful in 32%^9^.

### Maxillomandibular advancement (MMA)

MMA involves the creation of a LeForte 1 osteotomy to the maxilla and a bilateral split sagittal osteotomy to the mandible. This procedure was first described with simultaneously performed hyoid suspension as treatment for OSAS by Riley et al.^22^ in 1986. Holty & Guilleminault^8^ performed a meta-analysis in 2012 demonstrating a mean AHI decrease from 63.9/h to 9.5/h, *p* < 0.001, with a surgical success rate of 86% and cure rate of 43.2%. It was noted that younger age (45 years), lower preoperative weight and AHI, an increase in posterior airway space > 11.6 mm, a maxillary advancement > 10 mm and a mandibular advancement more than 11.3 mm improved the likelihood of success. Pirklbauer et al.^12^ systematic review and analysis of data recommends a grade of A to B with regard to levels of evidence based medicine.

### Rapid maxillary expansion (RME)/Surgicalfy-assisted rapid palatal expansion (SARPE)

An initial case report by Palmisano & Cistulli^34^, followed by a case series by Cistulli et al.^35^, has been performed on adults, and demonstrated an improvement in AHI from 19 ± 4/h to 7 ± 4/h. In this study, the patients presented with transverse maxillary insufficiency and the success rate was 90% and the cure rate was 70%^35^.

### Mandibular distraction osteogenesis

There is limited data on mandibular distraction osteogenesis as treatment for OSAS in adults. Li et al.^39^ performed mandibular distractions on five healthy adults and after 12 months, found a mean improvement in AHI from 49/h to 7/h. In this study, the distraction osteogenesis advanced the mandibles between 5.5 mm to 12.5 mm, with a mean of 8.1 mm.

### Tracheostomy

Valero & Alroy^18^ demonstrated the effectiveness of tracheostomy (tracheotomy) as treatment for obstructive sleep apnea in a patient with acquired micrognathia (1965). Holty & Guilleminault^1^ pooled nine studies and found that the apnea index decreased from 88.4/h to 0.5/h (*p* < 0.001), AHI during REM decreased from 63.8/h to 26.2/h (*p* < 0.001) and AHI during NREM decreased from 98.9/h to 21.6/h (*p* < 0.001). Despite the morbidity from the surgery, the patients who have had long term tracheostomies experienced a significant decrease in mortality66., 67.. Partinen et al.^66^ evaluated 198 patients treated with either a tracheostomy (N = 71) or weight loss (N = 127), and had no deaths after five years in the tracheostomy group and 14 deaths in those in the weight loss group. He et al.^67^ demonstrated that tracheostomy is equivalent to CPAP at 8 years, with no deaths in these groups during the study period.

## DISCUSSION

Despite several different types of surgeries used on an annual basis to treat obstructive sleep apnea syndrome (~35,000/year), the surgeries most often performed in the United States are palatal surgeries, at an estimated 33,000/year^68^. Other procedures described herein are much less frequently performed, but include hypopharyngeal surgeries (~6500/year), genioglossus advancement (~5100/year), maxillomandibular advancement (~1400/year) and hyoid suspension (~675/year)^68^. The experience at Stanford has seen the development or application of several techniques for treatment of OSAS to include maxillomandibular advancement, genioglossus advancement, hyoid suspension and radiofrequency ablation, each of which have been modified or expanded by other colleagues around the country and around the world21., 22., 36.. Innovation and technology are also providing improved outcomes as demonstrated by the application of radiofrequency, coblation and transoral robotic surgery33., 36.. In addition to the challenges of keeping up with the current evidence and technology, there will be patients who fail sleep surgery. In order to maximize success and cure rates, multiple procedures are often necessary. Some patients may fail soft tissue surgeries and may be candidates for either maxillomandibular advancements or tracheostomies1., 8..

## CONCLUSION

There is a large amount of variability in outcomes for sleep surgeries, however, in order to maximize success and cure rates, multiple procedures are most often necessary. Technology is advancing, and sleep surgeons must keep up with current literature and participate in multi-disciplinary care in order to maximize outcomes for our patients.
